# CANDI: an R package and Shiny app for annotating radiographs and evaluating computer-aided diagnosis

**DOI:** 10.1093/bioinformatics/bty855

**Published:** 2018-10-10

**Authors:** Marcus A Badgeley, Manway Liu, Benjamin S Glicksberg, Mark Shervey, John Zech, Khader Shameer, Joseph Lehar, Eric K Oermann, Michael V McConnell, Thomas M Snyder, Joel T Dudley

**Affiliations:** 1 Department of Genetics and Genomic Sciences, Icahn School of Medicine at Mount Sinai, New York, NY, USA; 2 Institute for Next Generation Healthcare, Icahn School of Medicine at Mount Sinai, New York, NY, USA; 3 Verily Life Sciences LLC, South San Francisco, CA, USA; 4 Institute for Computational Health Sciences, University of California, San Francisco, CA, USA; 5 Department of Radiology, Icahn School of Medicine at Mount Sinai, New York, NY, USA; 6 Department of Medical Informatics, Northwell Health, Centre for Research Informatics and Innovation, New Hyde Park, NY, USA; 7 Department of Bioinformatics, Boston University, Boston, MA, USA; 8 Department of Neurological Surgery, Icahn School of Medicine at Mount Sinai, New York, NY, USA; 9 Division of Cardiovascular Medicine, Stanford School of Medicine, Stanford, CA, USA

## Abstract

**Motivation:**

Radiologists have used algorithms for Computer-Aided Diagnosis (CAD) for decades. These algorithms use machine learning with engineered features, and there have been mixed findings on whether they improve radiologists’ interpretations. Deep learning offers superior performance but requires more training data and has not been evaluated in joint algorithm-radiologist decision systems.

**Results:**

We developed the Computer-Aided Note and Diagnosis Interface (CANDI) for collaboratively annotating radiographs and evaluating how algorithms alter human interpretation. The annotation app collects classification, segmentation, and image captioning training data, and the evaluation app randomizes the availability of CAD tools to facilitate clinical trials on radiologist enhancement.

**Availability and implementation:**

Demonstrations and source code are hosted at (https://candi.nextgenhealthcare.org), and (https://github.com/mbadge/candi), respectively, under GPL-3 license.

**Supplementary information:**

Supplementary material is available at *Bioinformatics* online.

## 1 Introduction 

Computer vision algorithms have demonstrated success in many fields including medical radiology. Convolutional neural networks (CNNs) are a type of deep learning (DL) model that automatically learns image features and can be applied to several image-recognition tasks. Successful models are trained on the order of 100 000 training images acquired through multi-site efforts (Gulshan *et al.*, 2016; Ting *et al.*, 2017). In medicine, data collection and crowdsourcing are complicated by privacy and specialized training requirements.

Web-based medical image annotation tools have been described but kept proprietary to an institution (Mata *et al.*, 2016, 2017) and to specific crowdsourced projects (Cheplygina *et al.*, 2017; Maier-Hein *et al.*, 2014). LabelMe is a fully featured online tool designed for everyday images, but does not support sensitive data (Russell *et al.*, 2008). In clinical practice radiologists interpret images in the context of a patient’s previous image studies and non-image medical record data. There is a lack of annotation tools that provide multimodal patient data interfaces and can be deployed for collaborative work on sensitive data.

Algorithms designed for Computer-Aided Diagnosis (CAD) are frequently only evaluated in isolation, and studies evaluating human performance with and without CAD have had inconsistent results. Retrospective studies on engineered feature (not DL) CAD in clinical practice have found accuracy benefit (Kasai *et al.*, 2008), no accuracy benefit (Benedikt *et al.*, 2017), or a negative effect (Gilbert *et al.*, 2008). CAD enhancement of human interpretation has been studied in disparate experimental designs. Commercially available CAD tools have been tested in fully randomized studies (Gilbert *et al.*, 2008) and observational studies (Fenton *et al.*, 2007). Experimental algorithms have been tested in only one mode (see RCT Case Study below) (Kasai *et al.*, 2008), or over multiple sessions (double-crossover design) where one day a radiologist interprets images with CAD and several months later she interprets images without CAD (or vice versa, by randomization) (Benedikt *et al.*, 2017). RCTs are graded as stronger evidence than pseudorandomized or observational studies, but RCTs have only been done with commercially available CAD systems.

This manuscript introduces two open access computer-aided note and diagnosis interface (CANDI) web applications for collaboratively addressing the annotation and evaluation barriers to translating DL. The CANDI radiograph annotation dashboard (CANDI-RAD) app provides multimodal patient and image data to obtain training and testing data, and the CANDI-CAD evaluation app facilitates randomized controlled trials (RCTs) on human enhancement with algorithms.

## 2 Implementation

CANDI is distributed as an R package with web interfaces implemented as Shiny applications and modules which generate html and javascript browser-based dashboards. CANDI’s modules handle user input and render an image or all the images from a selected case, along with patient metadata. Additional modules for annotation graphically summarize a user’s entry records, and evaluation modules support CAD utilities (e.g. searching for similar images) and queue randomization. The package includes metadata from the public OpenI chest X-ray database (Demner-Fushman *et al.*, 2016) to demonstrate multimodal dashboards [images are separately available from the CC-NC-ND licensed openI database (https://openi.nlm.nih.gov/)].

We use third-party packages to support data input and output. The European Bioinformatics Institute package EBImage reads and renders standard biomedical image formats from disk or URL. The googlesheets package saves user input to the cloud for de-identified annotation storage. CANDI builds on these individual packages by providing Shiny modules so users can compile an interface suited for their study context (Badgeley *et al.*, 2016).

Demonstration apps and user instructions are available at candi.nextgenhealthcare.org, which is hosted by a Nginx cloud server running Ubuntu. The CAD utilities were generated with several variations of Convolutional Neural Networks (CNNs) to predict disease status and localization and similar image search (further discussed in the Supplementary Material). The similar search module uses CNN image embeddings to compute the Euclidean distance between a test radiograph and all designated historical radiographs.

## 3 Case studies

### 3.1 Annotation

**Fig. 1.**
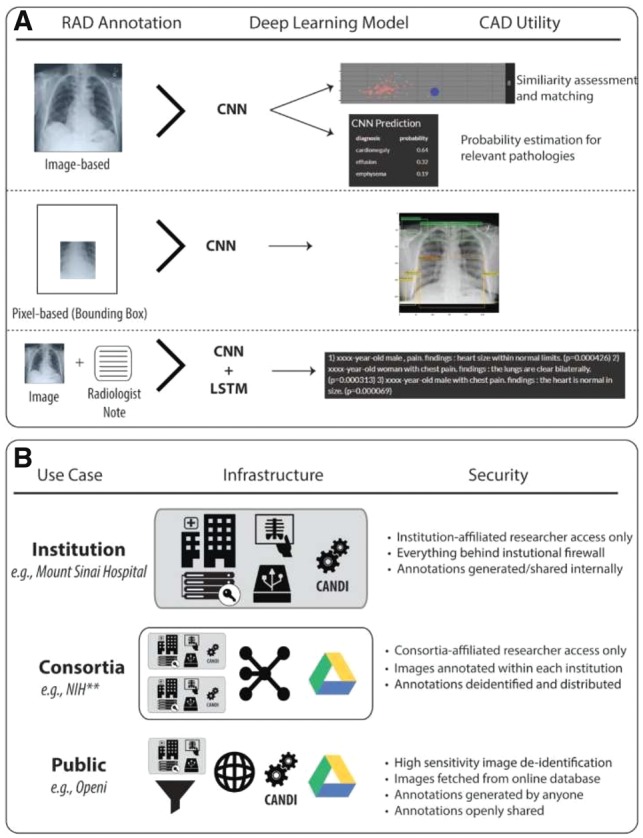
**Annotation modalities and distinct uses.** (**A**) The CANDI radiograph annotation (RAD) and computer-aided diagnosis (CAD) applications provide human-algorithm interfaces to generate training annotations and evaluate the subsequent models. Different annotation data modalities provide training data for distinct deep learning model utilities. We use convolutional neural networks (CNNs) to generate predictions in CANDI-CAD. (**B**) Various input/output systems are set up that conform to the security needs of different types of users

The CANDI training data generation app (candi.nextgenhealthcare.org/rad_institution) collects annotations for three supervised learning problems: (i) disease classification, (ii) image segmentation and (iii) image captioning. Each of these can be used to train a different implementation of a CNN (see Fig. 1). To adjudicate the gold-standard disease status, radiologists should use the multimodal app (candi.nextgenhealthcare.org/rad_case) to benefit from contemporaneous images and patients’ clinical data.

### 3.2 Evaluation randomized control trial

We implement CANDI-CAD to measure how users interpret radiographs under different assistance modes: concurrent and second-reader. In concurrent mode, the user receives algorithm support during the entire case interpretation, whereas in second-reader mode, algorithm support is only provided after the user formulates an initial unaided impression.

Rigorous evaluation of new algorithms requires CAD software to be integrated into image database systems (Matsumoto *et al.*, 2013). CANDI-CAD enables experimental algorithms to be incorporated into image interpretation dashboards with randomized availability of CAD utilities. The demonstration at (candi.nextgenhealthcare.org/cad) uses three DL utilities: (i) Image similarity search, (ii) whole image classification and (iii) image bounding-box localization (see Fig. 1). Image queue order and CAD mode are fully randomized to facilitate a 2-arm RCT in one session.

## 4 Conclusion

CANDI aims to ease the translation of CAD algorithms to medical imaging by facilitating collaborative image annotation and randomized clinical evaluation. CANDI-RAD facilitates distributed annotation with a multimodal interface for patient context, which reflects clinical practice and allows radiologists to produce gold-standard data. CANDI-CAD facilitates randomized clinical trials to rigorously evaluate CAD augmentation of radiologists’ performance. Different data input/output interfaces can be used to apply CANDI to sensitive or public medical image data.

## Funding 

This work was supported by Verily Life Sciences, LLC as part of the Verily Academic Partnership with Icahn School of Medicine at Mount Sinai and by the National Institutes of Health, National Center for Advancing Translational Sciences (NCATS), Clinical and Translational Science Award [UL1TR001433-01] to J.T.D.

*Conflict of Interest:* JTD has received consulting fees or honoraria from Janssen Pharmaceuticals, GlaxoSmithKline, AstraZeneca and Hoffman-La Roche. JTD is a scientific advisor to LAM Therapeutics and holds equity in NuMedii, Ayasdi and Ontomics. JL currently works for Merck in addition to his adjunct professor role at Boston University.

## Supplementary Material

Supplementary Data
