# Supplementary material for: CANDI: an R package and Shiny app for annotating radiographs and evaluating computer-aided diagnosis
Source: Bioinformatics. 2018 Oct 10;35(9):1610–2. doi: 10.1093/bioinformatics/bty855 (PMC6499410; doi:10.1093/bioinformatics/bty855)
Supplement: Supplementary Data [file bty855_online_supplement.docx]

Supplementary Methods

Application Image Dataset

The open source IU dataset was obtained from the OpenI repository (<https://openi.nlm.nih.gov/gridquery.php?coll=cxr>), containing 7,470 images from 3,999 studies. Images were retrieved through the OpenI RESTful API, and corresponding metadata and manually generated labels were parsed from DICOM files and JSONs [(Demner-Fushman et al., 2016)](https://paperpile.com/c/oadjbM/zujXC).

Disease Labels

The OpenI chest x-ray dataset curators reviewed every radiology impression note and annotated it with Medical Subject Heading (MeSH) terms.  Their manually acquired labels were used for model training and evaluation [(Demner-Fushman et al., 2016)](https://paperpile.com/c/oadjbM/zujXC).

Model Training Datasets

Additional datasets aggregated only for training deep learning models (See Supplemental Table 1).

**Supplemental Table 1:** Chest X-Ray Dataset Sources.

| Dataset | Data Availability | Samples | Source |
| --- | --- | --- | --- |
| Indiana University (IU) | Public | 7,399* | https://openi.nlm.nih.gov/services.php?it=xg |
| National Institutes of Health (NIH) | Public | 112,077* | https://nihcc.app.box.com/v/ChestXray-NIHCC |
| Mount Sinai Health System (MSHS) | Private** | 48,902* | Primarily inpatient portable radiographs from Mount Sinai Hospital (MSH), but also includes PA/lateral radiographs from MSH and other sites in MSHS. |

* after exclusions (see supplementary methods)

** compulsatory per our Institutional Review Board agreement

Deep Learning Methods

Unsupervised Principal Component Analysis for Similarity Search

To obtain the clustering, we removed the final softmax and penultimate fully-connected layers to obtain 2,048 scalar features. Principal component analysis was then run on these features across the full dataset and data points were projected into the domain of the first two principal components (PC) domain to obtain the CANDI scatter plots.

Supervised Classification

We used the Inception v3 CNN image model architecture along with model weights that were pre-trained on ImageNet natural images (Szegedy et al., 2015).  We use the CNN to infer all but the final layer of neural net activations for each image.  At the penultimate layer, the model transforms 2,048 3D tensors to scalars. Each scalar value captures a unique aspect of the input image, so we call this embedded collection of feature measurements the “image phenotype”.

To detect the presence of an imaging indication in an image, we trained a logistic regression model that ingested the image phenotype, patient demographics (age, sex), and the projection of the radiograph (AnteroPosterior, PosteroAnterior, or Lateral).  For datasets with >100 missing values of any of these variables, we used an explicit “missing” value as a factor level for the variable. When less than 100 images from a dataset were missing a variable, we excluded those images from the study.

This left us with a combined 168,378 radiographs from IU, MSH, and NIH as elaborated in Supplemental Table 1.

We used 10-fold cross-validation (CV) to iteratively train models, and evaluate them on the fold’s hold-out data that wasn’t used to fit the model in that fold.  The R statistical language caret package was used to ensure the same CV splits were applied to the models trained for all 9 imaging indications. Images were grouped by study or patient before splitting to avoid having the same patient’s images in both train and test data splits.  The performance metrics and ROC curves were computed based on the hold-out data of each fold.

Supervised Segmentation with Bounding Boxes

Bounding box learning is done via an objection detection architecture called SSD300 (Liu et al., 2015).  SSD300 examines many region proposals, performs multibox aggregations, and extensive negative example sampling to balance out negative and positive boxes.  To assess overlap between the true and predicted bounding boxes on the NIH dataset, we use the Mean Average Precision (MAP) metric.

Supplemental Results

Classification

CNN models trained on whole images generate probabilities to rank each disease. The classification performance statistics and receiver operator characteristic are shown for each disease that was annotated in all three datasets (see Supplemental Table 2 for performance statistics and Supplemental Figure 1 for ROC curves).  Across all disease indications, the classification models’ Area Under the Curve for the ROC curve (AUC) and Precision-Recall Curve (AUPRC) were 0.78+/- 0.10 and 0.81+/-0.13, respectively (mean +/- SD).

**Supplemental Table 2:** Whole-image classification performance for 9 cardiothoracic pathologies.  AUROC=area under ROC, AUPRC=area under precision-recall curve, Acc=accuracy, Sens=sensitivity, Spec=specificity, PPV=positive predictive value, NPV=negative predictive value, FPR=false positive rate (=1-spec)

| Indication | AUROC | AUPRC | Acc | Sens | Spec | PPV | NPV | FPR |
| --- | --- | --- | --- | --- | --- | --- | --- | --- |
| Consolidation | 0.89 | 0.64 | 0.87 | 0.66 | 0.92 | 0.66 | 0.92 | 0.08 |
| Pneumonia | 0.87 | 0.78 | 0.89 | 0.43 | 0.94 | 0.43 | 0.94 | 0.06 |
| Edema | 0.83 | 0.87 | 0.91 | 0.28 | 0.95 | 0.28 | 0.95 | 0.05 |
| Cardiomegaly | 0.83 | 0.77 | 0.86 | 0.40 | 0.92 | 0.40 | 0.92 | 0.08 |
| Hernia | 0.82 | 0.99 | 0.99 | 0.04 | 1.00 | 0.04 | 1.00 | 0.00 |
| Effusion | 0.78 | 0.66 | 0.80 | 0.51 | 0.87 | 0.51 | 0.87 | 0.13 |
| Atelectasis | 0.76 | 0.70 | 0.81 | 0.46 | 0.88 | 0.46 | 0.88 | 0.12 |
| Nodule | 0.64 | 0.94 | 0.92 | 0.07 | 0.96 | 0.07 | 0.96 | 0.04 |
| Emphysema | 0.62 | 0.97 | 0.96 | 0.06 | 0.98 | 0.06 | 0.98 | 0.02 |


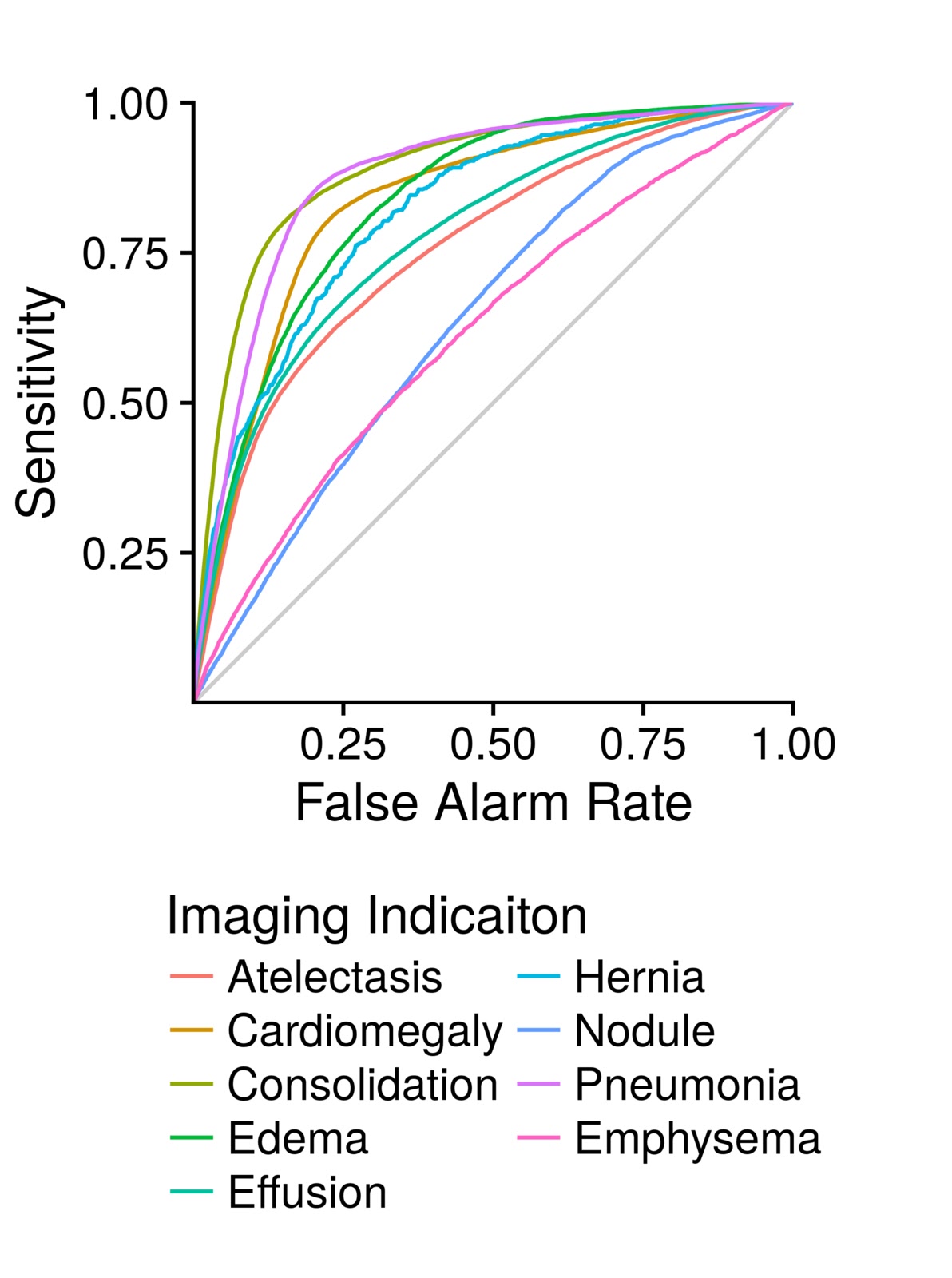


**Supplemental Figure 1:** Receiver Operator Characteristic Curve for Whole Image Classifiers of 9 Cardiothoracic Imaging Indications.  ImageNet pre-trained CNNs embedded 168,368 radiographs from the IU, NIH, and MSH datasets into image phenotypes, and a logistic regression was trained via 10-fold train-test splits to predict each imaging indication given the image phenotype and patient metadata (age, gender, x-ray projection).  Models performed variably depending on the imaging indication. The best models predicted consolidation and pneumonia (AUROC 0.89 and 0.88), and the worst predicted nodule and emphysema (AUROC 0.64 and 0.62).

Segmentation

The localization CNN produces bounding boxes to highlight portions of the input image concerning for each pathology.  This utility has a Mean Average Precision (MAP) score of 2.1%, with its best performance on Cardiomegaly at an AP of 9.1% and worst score on Nodules with an AP of 0.01% (see Supplemental Table 3).

**Supplemental Table 3:** Bounding-box Localization Performance Scores for 9 Cardiothoracic Imaging Indications.

| **Disease Label** | **Average Precision (AP) %** |
| --- | --- |
| Atelectasis | 1.03 |
| Cardiomegaly | 9.09 |
| Effusion | 0.51 |
| Infiltrate | 3.03 |
| Mass | 0.07 |
| Nodule | 0.01 |
| Pneumonia | 2.02 |
| Pneumothorax | 1.09 |
| Overall Mean | 2.11 |

Supplemental Discussion

Data Security Implementations For Different Collaboration Types

Medical image data is considered protected health information and cannot be shared without complex deidentification processes, while the annotations are structured data that can be easily deidentiifed.  For public (deidentified) image datasets, CANDI can directly fetch data ad hoc via url {{candi.nextgenhealthcare.org/rad_public}}. For use by a consortia, users at multiple sites may have images and patient metadata that cannot be shared outside their site.  In this setting CANDI can be deployed at each site; users can temporary upload images and annotate; and then deidentified annotations can be aggregated {{candi.nextgenhealthcare.org/rad_consortia}}. For private data, a server securely placed behind an institutional firewall can locally house image and annotation data and run CANDI to only allow internal users access.

Limitations on Customizability and Data Privacy

The Shiny framework can provide a communal image annotation and CAD evaluation platform, but has notable limitations.  For customization, familiarity with the R statistical language and R shiny web framework is required. Also, to ensure HIPAA compliance, researchers must either deploy CANDI to a private Shiny Server with separate access control or subscribe to a paid Shiny Server Profession subscription (which allows embedded authorization, visit https://www.rstudio.com/products/shiny-server-pro).

References

[Demner-Fushman, D., Kohli, M. D., Rosenman, M. B., Shooshan, S. E., Rodriguez, L., Antani, S., … McDonald, C. J. (2016). Preparing a collection of radiology examinations for distribution and retrieval. *Journal of the American Medical Informatics Association: JAMIA*, *23*(2), 304–310.](http://paperpile.com/b/oadjbM/zujXC)

[Liu, W.*,* Anguelov, D., Erhan, D., Szegedy, C., Reed, S., Fu, C., Berg, A.C. (2015). SSD: Single Shot MultiBox Detector. *arXiv [cs.CV]*.](http://paperpile.com/b/xAIqkJ/8Nx3)

[Szegedy, C., Vanhoucke, V., Ioffe, S., Shlens, J. & Wojna, Z. (2015). Rethinking the Inception Architecture for Computer Vision. *arXiv [cs.CV]*.](http://paperpile.com/b/xAIqkJ/IyFb4)
